# AAV‐mouse DNase I sustains long‐term DNase I expression in vivo and suppresses breast cancer metastasis

**DOI:** 10.1096/fba.2024-00114

**Published:** 2024-08-30

**Authors:** Melanie Herre, Kalyani Vemuri, Jessica Cedervall, Stefanie Nissl, Falk Saupe, Jacob Micallef, Henrik Lindman, Casey A. Maguire, George Tetz, Victor Tetz, Anna‐Karin Olsson

**Affiliations:** ^1^ Department of Medical Biochemistry and Microbiology, Biomedical Center Uppsala University Uppsala Sweden; ^2^ Belgian Volition SRL Parc Scientific Créalys Isnes Belgium; ^3^ Department of Immunology, Genetics and Pathology, Rudbeck Laboratory Uppsala University Uppsala Sweden; ^4^ Department of Neurology Harvard Medical School, Massachusetts General Hospital Boston Massachusetts USA; ^5^ Molecular Neurogenetics Unit Massachusetts General Hospital Charlestown Massachusetts USA; ^6^ CLS Therapeutics New York New York USA; ^7^ Human Microbiology Institute Department of Systems Biology New York New York USA

**Keywords:** AAV, breast cancer, DNase I, metastasis, NETs, Neutrophilextracellular traps

## Abstract

Neutrophil extracellular traps (NETs) have been implicated in the pathology of various inflammatory conditions. In cancer, NETs have been demonstrated to induce systemic inflammation, impair peripheral vessel and organ function and promote metastasis. Here we show that the plasma level of NETs is significantly higher in patients with metastatic breast cancer compared to those with local disease, or those that were considered cured at a 5‐year follow‐up, confirming NETs as interesting therapeutic targets in metastatic breast cancer. Administration of DNase I is one strategy to eliminate NETs but long‐term treatment requires repeated injections and species‐specific versions of the enzyme. To enhance administration and therapeutic efficacy, we have developed an adeno‐associated virus (AAV) vector system for delivery of murine DNase I and addressed its potential to counteract cancer‐associated pathology in the murine MMTV‐PyMT model for metastatic mammary carcinoma. The AAV vector is comprised of capsid KP1 and an expression cassette encoding hyperactive murine DNase I (AAV‐mDNase I) under the control of a liver‐specific promotor. This AAV‐mDNase I vector could support elevated expression and serum activity of murine DNase I over at least 8 months. Neutrophil Gelatinase‐Associated Lipocalin (NGAL), a biomarker for kidney hypoperfusion that is upregulated in urine from MMTV‐PyMT mice, was suppressed in mice receiving AAV‐mDNase I compared to an AAV‐null control group. Furthermore, the proportion of mice that developed lung metastasis was reduced in the AAV‐mDNase I group. Altogether, our data indicate that AAV‐mDNase I has the potential to reduce cancer‐associated impairment of renal function and development of metastasis. We conclude that AAV‐mDNase I could represent a promising therapeutic strategy in metastatic breast cancer.

## Graphical abstract

Tumor‐secreted factors induce the formation of intravascular neutrophil extracellular traps (NETs), contributing to tumor‐associated complications such as organ dysfunction and metastasis. We have developed an adeno‐associated virus (AAV) vector expressing species‐specific murine DNase I (mDNaseI), an enzyme that can degrade NETs. Systemic DNase I activity was elevated over at least 8 months following vector injection. Tumor‐bearing mice treated with AAV‐mDNase I exhibited improved kidney function and a reduced incidence of metastasis, indicating that this could be a potential therapeutic strategy.

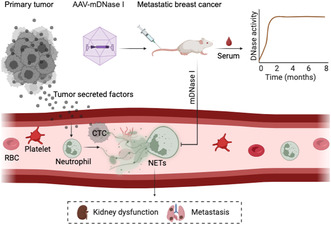

## 1 INTRODUCTION

Neutrophil extracellular traps (NETs) are formed when neutrophils externalize decondensed chromatin mixed with proteins from their azurophilic granules such as neutrophil elastase (NE) or myeloperoxidase (MPO). NETs can form in response to an infection, to help the host to contain the infectious agent and prevent further spread. However, formation of NETs comes at a price since they also exert cytotoxic effects due to exposed histones and high proteolytic activity and can damage both endothelium and epithelium at the site where they form.^1^, ^2^, ^3^ Neutrophils can be triggered to form NETs not only during infections, but also during sterile inflammation, and can contribute to the pathology of cancer and other inflammatory conditions.^4^ Cancer‐associated death is primarily connected to systemic effects such as thrombosis, distant organ failure and metastasis.^5^ Our previous studies show that NETs form in the circulation of mice with mammary carcinoma, where they impair vascular perfusion, cause systemic inflammation and upregulation of biomarkers for kidney and heart dysfunction.^6^, ^7^, ^8^ In addition, a promoting role of NETs in breast cancer metastasis has been demonstrated in a number of studies. By immunofluorescence staining of primary tumor tissue and matched metastases from breast cancer patients, Park et al found that the presence of NETs was associated with a more aggressive subtype of breast cancer.^9^ Plasma levels of NE‐DNA complexes, a readout for NETs, are also reported to increase with more advanced stages of breast cancer.^10^ Various mechanisms for how NETs could promote breast cancer metastasis in preclinical mouse models have been suggested. For example, it has been shown that the protease cathepsin C, secreted from tumor cells, plays a central role in recruiting neutrophils to metastatic niches and to induce NET formation, which in turn promotes lung colonization of breast cancer.^11^ Neutrophils prone to form NETs have been connected to establishment of premetastatic niches during breast cancer metastasis.^12^, ^13^ NETs have also been shown to have the capacity to awaken dormant breast cancer cells in the lung by proteolytic processing of the extracellular matrix protein laminin, resulting in stimulation of proliferation of the dormant cancer cells.^14^ A recently published paper shows that chemotherapy can induce NET formation, which can confer treatment resistance and invasive behavior via TGF‐beta release and activation from its latent form stored in the extracellular matrix.^15^

Considering the high mortality connected to breast cancer metastasis, NETs constitute an interesting therapeutic target to prevent or reduce spread of tumor cells. Several studies have shown that NETs can be degraded using systemic DNase I administration.^4^ In preclinical studies, daily injections of bovine DNase I, due to its short half‐life after intravenous injection, is commonly used for this purpose. For clinical development of DNase I as a NET‐targeting approach, it is desirable to reduce the number of injections, both from the patient perspective and to reduce the cost. It would likely also be beneficial to keep the DNase I at a steady high level, without fluctuation over the day. One strategy to achieve this is through adeno‐associated virus (AAV)‐mediated expression of DNase I. To enable evaluation of this approach in mouse models, we have generated an AAV‐vector expressing murine hyperactive DNase I (AAV‐mDNase I). The AAV vector is comprised of capsid KP1 and an expression cassette encoding hyperactive murine DNase I (AAV‐mDNase I) under the control of a liver‐specific promotor. The hyperactive version of DNase I is achieved by three point mutations, which enhance activity and impair inhibition by salt and actin, as previously described.^16^ In the current study we show that this AAV‐mDNase I vector could sustain high DNase I activity in serum of mice over several months after only one intravenous injection. Moreover, cancer‐associated upregulation of the kidney dysfunction biomarker NGAL was suppressed by the AAV‐mDNase I administration. In addition, the proportion of mice that developed lung metastasis was reduced in the group that received AAV‐mDNase I compared to a control group receiving an empty vector (AAV‐null). Our data show that it is possible to achieve a long‐term increase of DNase I activity in the circulation through AAV‐mediated expression and that this approach has the potential to counteract cancer‐associated pathology.

## 2 MATERIALS AND METHODS

### 2.1 Mice

Animal work was approved by the local ethics committee (dnr: C129/15;14613/2020) and performed according to the United Kingdom Coordinating Committee on Cancer Research guidelines for the welfare of animals in experimental neoplasia. For this study female MMTV‐PyMT transgenic mice (FVB/n background) were used. The MMTV‐PyMT breast cancer model is characterized by spontaneous development of adenocarcinomas of all mammary epithelia by 8–10 weeks of age, with a high incidence of pulmonary metastases. Littermates that lack the transgene were used as healthy controls. In the AAV‐pilot and the AAV‐tumor study, groups of *n* = 3 and *n* = 10 mice were included respectively. Discrepancies in animal number in the results are due to poor RNA quality, or loss of animals before the experimental endpoint.

### 2.2 Bovine DNase I injection

Mice were treated by daily intraperitoneal injections of bovine DNase I (10 U in PBS, EN0521; ThermoFisher Scientific) during 3 days or as otherwise indicated. Serum was collected as described below.

### 2.3 AAV expression plasmid construction

The plasmid, pAAV‐ApoEHCR enhancer‐hAAT promoter‐mDNase I (hyperactive)‐WPRE Xinact (AAV‐mDNaseI) was constructed as previously described with some minor modifications.^17^ The cDNA of the transgene expression cassette for mCLS‐014 was synthesized by GenScript (Piscataway, NJ, USA). A 5′ EcoRI site and a 3′ HindIII site were added to the sequence of mCLS‐014. The cassette consists of (a) apolipoprotein E‐hepatic control region (APOE‐HCR enhancer) and human alpha‐1‐antitrypsin (hAAT) promoter, (b) a Kozak sequence, (c) mouse hyperactive, salt and actin resistant DNase I‐variant having the natural signal sequence,^16^ (d) a woodchuck hepatitis virus posttranscriptional element (WPRE) with the X protein coding region, which was inactivated by mutating at the start codon [WPRE‐X‐inactivated (woodchuck hepatitis virus posttranscriptional regulatory element with X protein inactivated)] (Figure 2B). The mCLS‐014 DNA was digested with EcoRI‐HF and HindIII‐HF (New England Biolabs, Ipswich, MA, USA). An AAV promoterless expression vector, pAAV‐MCS‐Promoterless providing a polyA signal (pA) and two flanking AAV2 inverted terminal repeats, ITRs, for the mCLS‐014 cassette (Cell Bio Labs Cat no. VPK‐411, San Diego, CA, USA) were used. Both the pAAV‐MCS Promoterless vector and mCLS‐014 cassettes were digested with EcoRI‐HF and HindIII‐HF, and ligated for 1 h at room temperature in a 3:1 insert to vector molar ratio. The ligated construct was transformed into SURE electrocompetent cells (Agilent Technologies, Santa Clara, CA, USA) following plating on LB agar ampicillin plates. Colonies growing on agar were selected and minipreps were prepared for screening. To determine the presence of expected bands, AAV2‐inverted terminal repeats (ITR) were digested with SmaI. Bacterial clones were selected when the expected bands were present in each of the SmaI, EcoRI‐HF, and HindIII‐HF digests. The whole‐plasmid sequencing of AAV‐mDNaseI plasmid was conducted (MGH DNA core) followed by purification using an endo‐free Gigaprep (Qiagen, Carlsbad, CA, USA) performed by Alta Biotech (Aurora, CO, USA). (Figure 2). For the control group we also engineered an AAV‐null which contained all components of AAV‐mDNase I plasmid with the exception that it was devoid of the mDNase I cDNA.

### 2.4 AAV production

AAV‐mDNase I vector was produced by Boston Children's Hospital Vector Core using large‐scale polyethylenimine (PEI) triple plasmid transfections of rep/cap plasmid encoding the KP1 capsid,^18^ AAV‐mDNase I plasmid with flanking ITRs, and adenovirus helper plasmid (pAd5) in adherent HEK293 cells with purification on an iodixanol density gradient via ultracentrifugation using standard protocols. The purified AAV‐mDNase I vector was resuspended in PBS supplemented with 35 mm NaCl and 0.001% Pluronic F68, followed by a quantitative PCR to determine the vector titer. After titration, all vectors were diluted to a titer of 1.0 × 10^13^ genome copies per mL (GC per mL). The purified AAV‐mDNase I vector was analyzed by SDS/PAGE. Three bands of 60, 72, and 90 kDa were observed in a ratio of ~1:1:10, which corresponds to the VP1‐3 proteins. AAV‐null contained all the components of the AAV‐mDNase I transgene expression cassette, with the exception that it was devoid of DNase I cDNA. AAV vectors were stored at −80°C until use.

### 2.5 Human plasma samples

Analysis of human samples complies with the Declaration of Helsinki and was approved by the Ethical Review Board in Uppsala (dnr 2020‐06019). Citrated plasma samples were obtained through the U‐CAN project (www.u‐can.uu.se).^19^ The samples were derived from breast cancer patients at diagnosis with primary early breast cancer (Stage I‐III, *n* = 30, median age 62 years) and breast cancer patients with de novo metastatic disease (Stage IV, *n* = 26, median age 61 years). The metastases were located in bone (*n* = 12), liver (*n* = 9), lymph nodes (*n* = 9), lung (*n* = 8), pleura (*n* = 2) and the gastrointestinal tract (*n* = 1). Half of the metastatic patients had metastasis in more than one location. Individuals that were cancer free at 5‐year follow‐up (*n* = 30), were included as healthy controls. The plasma samples were taken at diagnosis before initiation of any cancer treatment. Samples were stored at −80°C.

### 2.6 Blood sampling and preparation

For serum sampling, mice were put under a heating lamp for 5 min. A small cut was made in the lateral tail vein with a scalpel. A few drops of blood (max. 200 μL) were collected, centrifuged at 2000*g* for 10 min and the obtained serum was stored at −80°C. For plasma collection, mice were anesthetized with 2% Avertin in PBS by intraperitoneal injection and blood was collected by cardiac puncture using citrate (0.0169 M) as an anticoagulant. The blood was centrifuged for 15 min at 150*g* to remove leukocytes and erythrocytes, followed by a second centrifugation at 1100*g* to remove platelets.

### 2.7 Urine sampling and preparation

Mouse urine was collected by light palpation of the bladder, centrifuged for 5 min at 5000*g* and stored at −20°C. The aim was to collect urine from all mice at the time of AAV injection (0 week), after 6 weeks and after 8 weeks However, urine sampling from all mice at each time‐point proved to be challenging. Thus, we limited our quantification to the 0 and 8 weeks’ time‐points. Successful sampling was achieved for *n* = 5 mice in the AAV‐null group and *n* = 4 mice in the AAV‐mDNase I group.

### 2.8 ELISA for the detection of neutrophil extracellular traps

NETs, H3R8Cit‐DNA complexes, were detected using a NET ELISA Kit (Nu.Q H3R8Cit; Volition), following the instructions from the manufacturer. In brief, precoated plates were washed and 20 μL undiluted human plasma, standard or control was added together with 80 μL assay buffer and incubated for 2.5 h at room temperature. Wells were washed and 100 μL of the HRP‐labeled detection antibody were added and incubated for 1.5 h at room temperature. Wells were washed and 100 μL 3,3′, 5,5′–tetramethyl benzidine (TMB) substrate solution was added to each well. The colorimetric reaction was stopped after 20 min and the optical density was determined at 450 nm with a microplate reader.

### 2.9 ELISA for the detection of anti‐bovine DNase I antibodies

ELISA plates were coated with 10 μg/mL bovine DNase I (EN0521; Thermo Scientific) in PBS and wells were blocked with horse serum. Mouse serum samples were diluted 1:10 in PBS. Anti‐bovine DNase I antibodies were detected with the biotinylated goat anti‐mouse IgG antibody (BA‐9200; Vector Laboratories) and streptavidin‐conjugated HRP (SA‐5004; Vector Laboratories) both diluted 1:500. All incubations were conducted at 37°C. HRP activity was detected with TMB substrate (T8665; Sigma) and the optical density was determined at 650 nm with a microplate reader.

### 2.10 ELISA for the detection of nucleosomes

Nucleosomes were detected using a nucleosome ELISA Kit (Nu.Q H3.1 ELISA Assay RUO; Volition), following the instructions from the manufacturer. In brief, precoated plates were washed and 20 μL undiluted mouse plasma, standard or control was added together with 80 μL of assay buffer and incubated for 2.5 h at room temperature. Wells were washed and 100 μL of the HRP‐labeled detection antibody were added and incubated for 1.5 h at room temperature. Wells were washed and 100 μL TMB substrate solution was added to each well. The colorimetric reaction was stopped after 20 min and the optical density was determined at 450 nm with a microplate reader.

### 2.11 RNA expression analysis by qPCR

Mice were anesthetized with an intraperitoneal injection of 2% Avertin in PBS and subsequently sacrificed by cervical dislocation. Organs were dissected and snap‐frozen using isopentane on dry ice. RNA was extracted using the RNeasy Midi kit (75142; Qiagen) and the iScript cDNA synthesis kit (1708891; Bio‐Rad) was used to prepare cDNA according to the manufacturer's instructions. The qPCR was performed using the KAPA SYBR FAST qPCR kit (KK4608; KAPA Biosystems). The RNA expression is presented relative to the expression of hypoxanthine‐guanine phosphoribosyltransferase (HPRT). Primer sequences used were:

HPRT forward primer: 5′‐CAAACTTTGCTTTCCCTGGT‐3′.

HPRT reverse primer: 5′‐TCGAGAGGTCCTTTTCACC‐3′.

mDNase I forward primer: 5'‐ATC GGG ACA AAC CTG ACA CC‐3′.

mDNase I reverse primer: 5′‐TTT CCA CAG GGT TCA CAG CC‐3′.

### 2.12 Single radial enzyme‐diffusion assay

A DNA solution containing 55 μg/mL DNA from salmon testes (15632‐011; Invitrogen) and manganese assay buffer (20 mM Tris–HCl pH 7.8; 10 mM MnCl_2_; 2 mM CaCl_2_; 2* SYBR Safe (S33102; Invitrogen)) was prepared and heated at 50°C for 10 min. The DNA solution was mixed with an equal volume of 2% ultra‐pure agarose (16500100; Invitrogen) in 0.5× TBE buffer. The mixture was poured onto a microscope slide (06314‐CC; Histolab) and stored at room temperature until solidification. Two microlitre of undiluted mouse serum was added to 1 mm wells. Bovine DNase I (EN0521; Thermo Scientific) was used as a standard. The samples were incubated in a humidified chamber for 4 h at 37°C. DNA fluorescence was recorded with a fluorescence scanner and the area of the dark circle created by DNA digestion was quantified as a measure for the DNase enzyme activity.

### 2.13 Liver enzyme analysis

Serum liver enzymes alanine aminotransferase (ALT), aspartate aminotransferase (AST) and alkaline phosphatase (ALP) were analyzed with Architect c4000 automatic biochemistry analyzer (AbbottDiagnostics) with reagents from Abbott Diagnostics (Figure 2) or with Beckman Coulter DxC 700 AU automatic biochemistry analyzer (Beckman Coulter) with reagents from Beckman Coulter (Supplemental Figure 1—Data S1). The analyses were performed at the Clinical Pathology Laboratory at the Swedish University of Agricultural Sciences, Uppsala.

### 2.14 Histology

Mice were anesthetized with an intraperitoneal injection of 2% Avertin in PBS and subsequently sacrificed by cervical dislocation. The left lung lobe was put in 30% sucrose solution at 4°C overnight, embedded in OCT Cryomount (45830; Histolab, Askim, Sweden) and cryosectioned (10 μm) at four different levels with 200 μm distance. The sections were fixed for 10 min in ice cold methanol and stained with Mayers hematoxylin (01820; Histolab) and Eosin Y 0.2% (01650; Histolab). Images were taken and metastases were detected using the Leica DMi8 fluorescent microscope.

### 2.15 Western Blot

The NuPAGE system (Life Technologies) was used for protein separation. Equal volumes of urine were loaded on the gel. After protein transfer to an Immobilon‐FL membrane (IPFL00010; Millipore), the membrane was blocked with Odyssey blocking buffer (927‐40000; LI‐CORE Biosciences). Neutrophil Gelatinase‐Associated Lipocalin (NGAL) was detected with an anti‐Lipocalin‐2 antibody (ab63929; 1:500; abcam), that was incubated over night at 4°C. IRDye 800CW donkey anti‐rabbit was used as a secondary antibody (926,32213; 1:10000; LI‐COR Biosciences) that was incubated for 1 h at room temperature. Imaging was done using the Odyssey Infrared Imaging System 2.1 (LI‐COR Biosciences). Background fluorescence intensity was measured from a region of the membrane without any protein bands and subtracted from the NGAL band intensity. The corrected NGAL band intensity was then normalized to the intensity of a positive control sample run on the same gel.

### 2.16 Statistical analysis

Statistical analyses in the study were performed using GraphPad Prism 9 (GraphPad Software Inc.). The unpaired *t test* was used to compare two groups with numerical values. To determine whether there is an association between two categorical values, the Fisher's exact test was used. Error bars indicate the standard deviation and * is defined as *p* < 0.05, **p < 0.01, ***p < 0.001, ****p < 0.0001.

## 3 RESULTS

### 3.1 Administration of bovine DNase I generates an anti‐bovine DNase I response in immunocompetent mice

**FIGURE 1:**
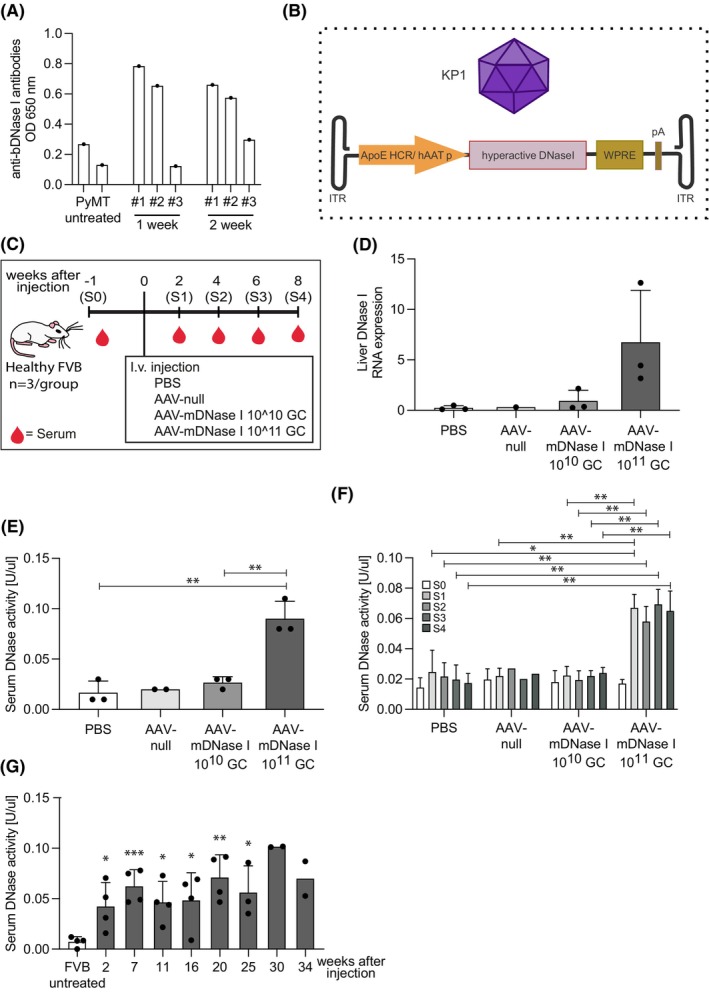
Elevated mDNase I liver expression and serum DNase activity after AAV‐mDNase I intravenous injection in tumor‐free mice. (A) ELISA for anti‐bovine DNase I (bDNase I) antibodies in MMTV‐PyMT mouse serum generated after 1 and 2 weeks of daily bDNase I intraperitoneal injections (*n* = 3) and in untreated MMTV‐PyMT mice (*n* = 2). (B) Schematic illustration of the AAV‐mouse DNase I vector. The engineered capsid, KP1, with high liver tropism was used for the study. ApoE HCR/hAATp = apolipoprotein E‐hepatic control region (APOE‐HCR enhancer) and human alpha‐1‐antitrypsin (hAAT) promoter. WPRE = woodchuck hepatitis virus posttranscriptional regulatory element. pA = polyadenylation signal sequence. We also engineered another vector, AAV‐null, which lacks the DNase I insert. (C) Schematic illustration of the AAV‐mDNase I pilot study. (D) Liver mDNase I expression was analyzed by qPCR 8 weeks after AAV injection (PBS *n* = 3; AAV‐null *n* = 1; AAV‐mDNase I 10^10^ GC *n* = 3; AAV‐mDNase I 10^11^ GC *n* = 3). (E) Serum DNase activity (S4) analyzed by the SRED assay (PBS *n* = 3; AAV‐null *n* = 2; AAV‐mDNase I 10^10^ GC *n* = 3; AAV‐mDNase I 10^11^ GC *n* = 3, PBS vs. AAV‐mDNase I 10^11^ GC *p* = 0.0037; AAV‐mDNase I 10^10^ GC vs. AAV‐mDNase I 10^11^ GC *p* = 0.0039). (F) Serum DNase activity over time (PBS *n* = 3; AAV‐null *n* = 3 (S0/1), *n* = 2 (S2‐4); AAV‐mDNase I 10^10^ GC *n* = 3; AAV‐mDNase I 10^11^ GC *n* = 3, *S1 PBS* vs. S1 AAV‐mDNase I 10^11^ GC *p* = 0.0122; S2 PBS vs. S2 AAV‐mDNase I 10^11^ GC *p* = 0.0096; S3 PBS vs. S3 AAV‐mDNase I 10^11^ GC *p* = 0.0033; S4 PBS vs. S4 AAV‐mDNase I 10^11^ GC *p* = 0.0048; S1 AAV‐null vs. S1 AAV‐mDNase I 10^11^ GC *p* = 0.0016; S1 AAV‐mDNase I 10^10^ GC vs. S1 AAV‐mDNase I 10^11^ GC *p* = 0.0020; S2 AAV‐mDNase I 10^10^ GC vs. S2 AAV‐mDNase I 10^11^ GC *p* = 0.0046; S3 AAV‐mDNase I 10^10^ GC vs. S3 AAV‐mDNase I 10^11^ GC *p* = 0.0015; S4 AAV‐mDNase I 10^10^ GC vs. S4 AAV‐mDNase I 10^11^ GC *p* = 0.0064). (G) Long‐term serum DNase activity in healthy FVB mice injected with AAV‐mDNase I and serum sampled at week 2, 7, 11, 16, 20 (*n* = 4), week 25 (*n* = 3), week 30, 34 (*n* = 2) (FVB untreated vs. 2 weeks after injection *p* = 0.0283; FVB untreated vs. 7 weeks after injection *p* = 0.0007; FVB untreated vs. 11 weeks after injection *p* = 0.0109; FVB untreated vs. 16 weeks after injection *p* = 0.0259; FVB untreated vs. 20 weeks after injection *p* = 0.0015; FVB untreated vs. 25 weeks after injection *p* = 0.0136). Statistical test used: Parametric two‐tailed unpaired *t*‐test. *p < 0.05, **p < 0.01, ***p < 0.001.

To address if DNase I administration can impair spontaneous lung metastasis in the MMTV‐PyMT (PyMT) model, an extended treatment period with DNase I is required. Since PyMT is an immunocompetent mouse model, there is a risk that the bovine DNase I will be recognized as foreign and induce a neutralizing antibody response after repeated injections. To investigate if this is indeed the case, we administered bovine DNase I daily for 2 weeks to PyMT mice and analyzed serum sampled after 1 or 2 weeks for anti‐bovine DNase I antibodies. As can be seen in Figure 1A, two out of three mice had anti‐bovine DNase I antibodies already after 1 week of treatment, which did not increase further after 2 weeks. The third mouse displayed a lower anti‐bovine DNase I response, which doubled after 2 weeks. From these experiments we conclude that it is not feasible to use bovine DNase I in immunocompetent mice for extended time periods.

### 3.2 AAV‐mediated expression and activity of murine DNase I could be sustained over several months

Since prolonged treatment with DNase I requires multiple injections of species‐specific DNase I, we turned to another strategy, expressing a murine hyperactive DNase I via an adeno‐associated virus (AAV) vector (AAV‐mDNase I; Figure 1B), under the control of a liver‐specific promoter. To determine if the AAV‐mDNase I vector could induce increased DNase I expression in the liver of injected mice, we performed a pilot study with four groups of healthy (FVB) mice receiving either PBS, AAV‐null or AAV‐mDNase I at two different doses (10^10^ genome copies (GC)/mouse and 10^11^ GC/mouse) via intravenous (iv) injection (Figure 1C). Serum was collected at start and then with 2 weeks intervals up to 8 weeks after injection. At the end of the study, liver RNA was prepared and the DNase I expression analyzed by qPCR (Figure 1D). Liver DNase I expression was clearly elevated in the mice receiving the higher dose of AAV‐mDNase I vector, while those receiving the lower dose showed a very modest increase in DNase I expression. DNase activity was measured in the serum samples at the end of the study (S4) using a single radial enzyme‐diffusion (SRED) assay (Figure 1E). In agreement with the liver expression data, DNase activity was significantly higher in serum from mice receiving the higher dose of the AAV‐mDNase I vector, while the other groups displayed DNase activity similar to control. We conclude from these data that the higher dose (10^11^ GC/mouse) of the AAV‐mDNase I vector was required to achieve elevated DNase activity in the circulation of injected mice and was therefore used in the following experiments.

The increased serum DNase activity could be detected already at the first sampling (S1) 2 weeks after AAV‐injection and appeared to have reached a maximum at this time point (Figure 1F). To address how long the elevated DNase activity could be sustained in the circulation after one dose of AAV‐mDNase I vector, healthy FVB mice were injected and serum sampled every month. DNase activity was significantly elevated compared to non‐injected mice for at least 25 weeks (Figure 1G). Although two mice were found dead toward the end of the analysis, the two remaining mice sustained expression at 34 weeks after injection (Figure 1G).

### 3.3 AAV‐mediated DNase I expression does not induce detectable liver toxicity

**FIGURE 2:**
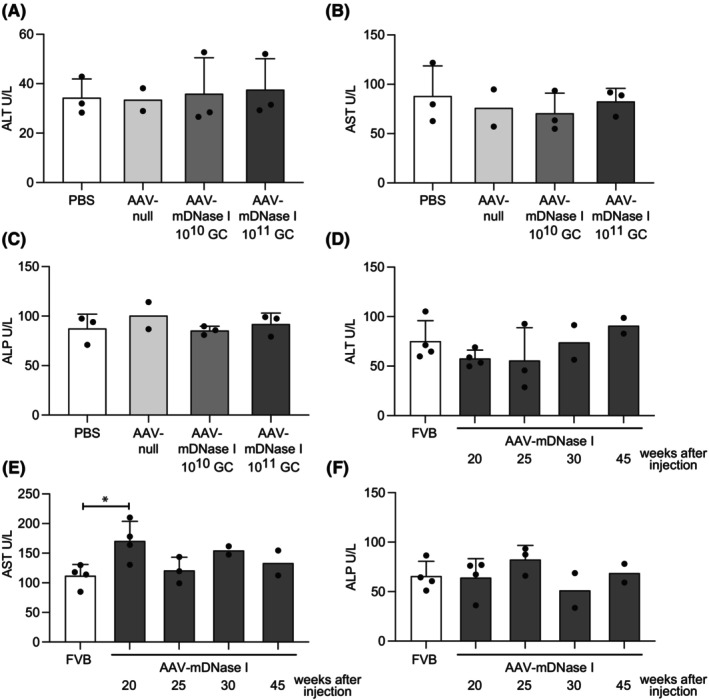
AAV‐mediated mDNase I administration in mice does not alter the expression of their liver enzymes. Analysis of (A) alanine aminotransferase (ALT), (B) aspartate aminotransferase (AST) and (C) alkaline phosphatase (ALP) in serum from healthy FVB mice 8 weeks after vector injection (S4) (PBS *n* = 3; AAV‐null *n* = 2; AAV‐mDNase I 10^10^ GC *n* = 3; AAV‐mDNase I 10^11^ GC *n* = 3). Analysis of long‐term (D) ALT, (E) AST and (F) ALP in healthy FVB mouse serum (FVB *n* = 4; AAV‐mDNase I 20 weeks after vector injection *n* = 4; AAV‐mDNase I 25 weeks after vector injection *n* = 3; AAV‐mDNase I 30 weeks after vector injection *n* = 2; AAV‐mDNase I 45 weeks after vector injection *n* = 2. The only statistically significant difference was the AST levels in mice injected with AAV‐mDNase I 20 weeks after vector injection, compared to untreated FVB mice (E, *p* = 0.0216). Statistical test used: Parametric two‐tailed unpaired *t‐*test. *p < 0.05.

To investigate if the AAV‐mDNase I or AAV‐null vector could potentially induce liver toxicity, serum samples from mice injected with either PBS, AAV‐null or the two different doses of AAV‐mDNase I were collected 8 weeks after injection and analyzed for the liver enzymes ALT, AST and ALP. As can be seen in Figure 2A–C, the serum concentration of these enzymes was similar in all four groups. Moreover, histological analysis of livers derived from the same mice did not reveal any morphological alterations (data not shown). To investigate if the long‐term expression of AAV‐mDNase I could induce liver toxicity we analyzed ALT, AST and ALP levels in healthy untreated FVB mice and FVB mice injected with AAV‐mDNase I and serum sampled the mice 20, 25, 30 and 45 weeks after vector injection (Figure 2D–F). No alterations of the liver enzymes were detected during this time. We could only see an increase in AST levels 20 weeks after vector injection, which was normalized in the following timepoints. Interestingly, the PyMT mice with mammary carcinoma showed significant changes in serum AST and ALP levels even without any treatment, indicating that the cancer itself can induce some degree of liver toxicity (Supplemental Figure 1A–C—Data S1).

### 3.4 Systemic administration of AAV‐mDNase I mediates elevated serum DNase activity in the MMTV‐PyMT breast cancer model

**FIGURE 3:**
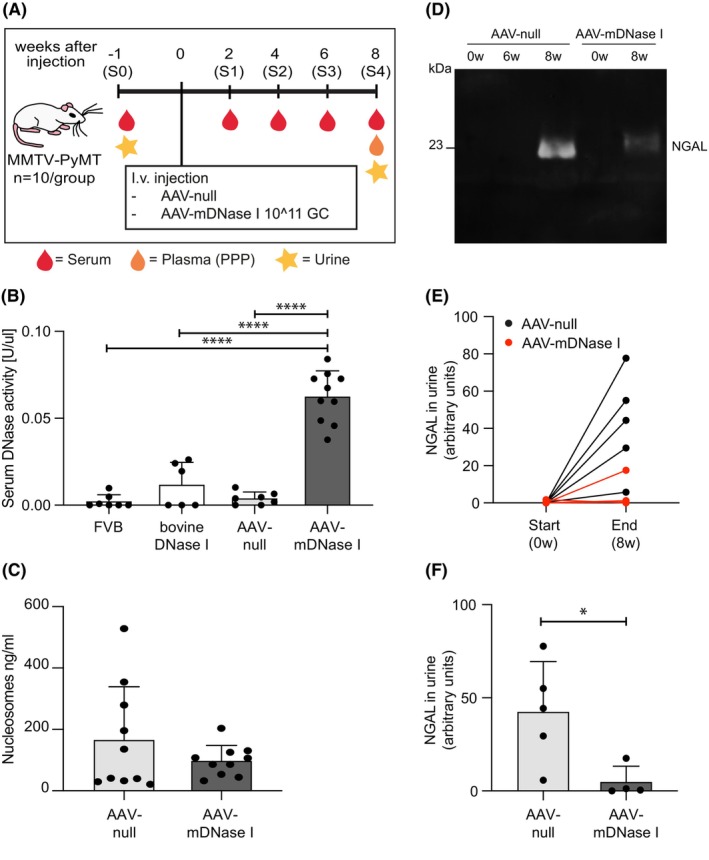
AAV‐mDNase I increases serum DNase activity and reduces secretion of the kidney dysfunction biomarker NGAL in MMTV‐PyMT mice. (A) Schematic illustration of the AAV‐mDNase I tumor study. (B) DNase activity measured by the SRED assay in serum from untreated healthy (FVB) mice, bDNase I‐injected mice serum sampled 4 h after injection, AAV‐null (S4) and AAV‐mDNase I (S4) injected MMTV‐PyMT mice (FVB *n* = 7; bDNase I *n* = 6; AAV‐null *n* = 7; AAV‐mDNase I *n* = 10, FVB vs. AAV‐mDNase I *p* < 0.0001; bDNase I vs. AAV‐mDNase I *p* < 0.0001; AAV‐null vs. AAV‐mDNase I *p* < 0.0001). (C) Nucleosome levels in mouse plasma from AAV‐null and AAV‐mDNase I mice sampled 8 weeks after AAV injection measured by ELISA (AAV‐null *n* = 10; AAV‐mDNase I *n* = 10). (D) Representative image of a Western blot for NGAL in urine from a mouse injected with AAV‐null and a mouse injected with AAV‐mDNase I at the start of the study (0 week), 6 weeks after vector injection (6 weeks, only AAV‐null) and at the end of the study (8 weeks). (E) Quantification of the Western blot for NGAL in urine from individual AAV‐null and AAV‐mDNase I‐injected MMTV‐PyMT mice before AAV injection (Start (0 week)) and 8 weeks after AAV injection (End (8 weeks)) (AAV‐null *n* = 5; AAV‐mDNase I *n* = 4). (F) Average NGAL‐level in the urine of AAV‐null and AAV‐mDNase I‐injected MMTV‐PyMT mice at the end of the study (End (8 weeks) in panel D) (AAV‐null *n* = 5; AAV‐mDNase I *n* = 4, AAV‐null vs. AAV‐mDNase I *p* = 0.0328). Statistical test used: Parametric two‐tailed unpaired *t*‐test. *p < 0.05, ****p < 0.0001.

To address if AAV‐mediated expression of murine DNase I could counteract breast cancer‐associated pathology, we administered AAV‐null or AAV‐mDNase I vector to 6‐weeks old PyMT mice. Before injection with AAV‐vectors, the PyMT mice were sampled for serum (S0) and subsequently every second week (S1–S4) (Figure 3A). At the end of the study (8 weeks after AAV injection) plasma was collected. In addition, urine was collected before injection of AAV‐vectors and at the end of the study (Figure 3A). Analysis of serum DNase activity again showed a significantly elevated level in mice receiving the AAV‐mDNase I vector compared to those injected with the AAV‐null vector or mice injected with 10 U of bovine DNase I (Figure 3B). Moreover, the serum DNase activity in PyMT mice that received the AAV‐null vector was not different from untreated PyMT‐negative littermates (FVB). As expected, the AAV‐mediated increase in DNase I expression was detected in the liver (DNase I expression is driven by a liver‐specific promoter), while expression in the kidney was not altered (Supplemental Figure 2A,B—Data S1). No DNase I expression could be detected in the heart and this was not affected by the AAV injection (data not shown). The amount of nucleosomes in plasma from the two groups of mice was analyzed after 8 weeks (S4) using the H3.1 ELISA^20^ and showed a trend toward lower levels in mice receiving the AAV‐mDNase I vector compared to those injected with the AAV‐null vector (Figure 3C).

### 3.5 AAV‐mediated DNase I activity suppresses breast cancer‐associated upregulation of the kidney dysfunction biomarker NGAL

NGAL constitutes an early biomarker for reduced vascular perfusion and hypoxia in kidney.^21^, ^22^ We have previously shown that there is an elevated expression of NGAL in renal tubules in PyMT mice and an increased secretion of NGAL in urine compared to healthy littermates.^7^ Targeting of NETs could counteract this elevated expression and secretion of NGAL.^7^ Analysis of the NGAL levels in urine by Western blot showed that the increased secretion over time seen in PyMT mice was significantly suppressed in mice that received the AAV‐mDNase I vector in contrast to those that received the AAV‐null vector (Figure 3D–F). We aimed to collect urine from all mice at the time of AAV injection (0 week), after 6 weeks and after 8 weeks, but did not succeed with sampling of all mice at each time‐point. Thus, we limited our quantification to the 0 week and 8 weeks’ time‐points with *n* = 5 mice in the AAV‐null group and *n* = 4 mice in the AAV‐mDNase I group (Figure 3E). In several of the mice that received the AAV‐mDNase I vector, there was hardly any increase in NGAL levels over the eight‐week period (Figure 3E). Overall, the NGAL levels in urine from mice that received AAV‐mDNase I was reduced by approximately nine times, compared to those that received AAV‐null (Figure 3F). These data indicate that the AAV‐mDNase I vector can counteract cancer‐associated renal dysfunction.

### 3.6 AAV‐mediated DNase I activity reduces the proportion of mice with spontaneous breast cancer lung metastasis

**FIGURE 4:**
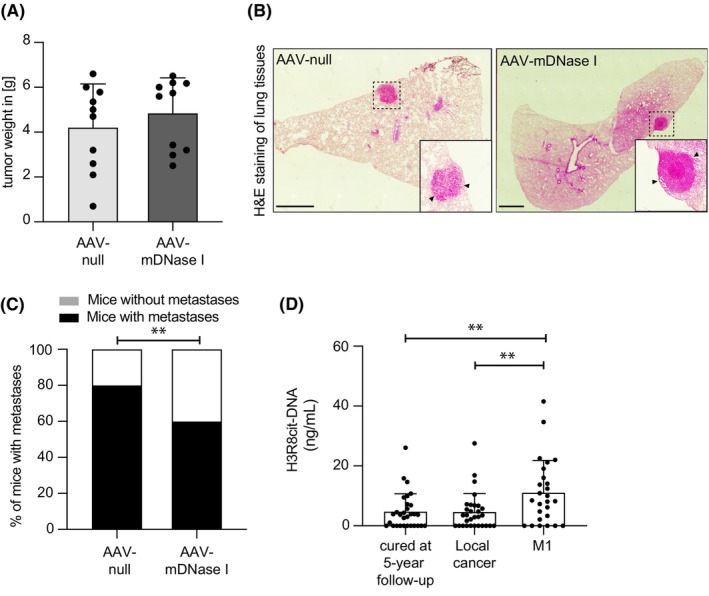
Metastatic breast cancer is associated with increased levels of circulating NETs and can be suppressed by AAV‐mDNase I. (A) Primary tumor weight at the end of the study (AAV‐null *n* = 10; AAV‐mDNase I *n* = 10). (B) Representative images of H&E‐stained lung sections from AAV‐null and AAV‐mDNase I injected mice. Arrowheads in the magnified insets indicate metastases. Scale bar: 1 mm. (C) Percentage of mice that developed lung metastasis among the AAV‐null and AAV‐mDNase I injected mice. The data was derived by complete sectioning of the lung tissue and H&E staining and analysis of four levels in each individual (AAV‐null *n* = 10; AAV‐mDNase I *n* = 10, AAV‐null vs. AAV‐mDNase I *p* = 0.0032). (D) ELISA for H3R8cit‐DNA complexes in plasma of breast cancer patients (cured at 5‐year follow‐up *n* = 30; local cancer *n* = 30; metastatic disease (M1) *n* = 26, cured at 5‐year follow‐up vs. M1 *p* = 0.0081; cured at 5‐year follow‐up vs. local cancer *p* = 0.9566; M1 vs. local cancer *p* = 0.0076). Statistical test used: (A, D) parametric two‐tailed unpaired *t‐*test; (C) Fisher's exact test. **p < 0.01.

To address if AAV‐mediated expression of hyperactive DNase I could counteract spontaneous breast cancer lung metastasis, we administered AAV‐null or AAV‐mDNase I vector to 6‐weeks old PyMT mice. Eight weeks later, when the mice had reached 14 weeks of age, primary tumors were dissected and weighed and lungs were preserved for histological analyses. Three mice in the AAV‐null group were sacrificed 2 weeks before the intended time due to the early development of large primary tumors. At the last day of the experiment, there was no difference in primary tumor weight between the two groups (Figure 4A). The presence of metastases was analyzed in hematoxylin and eosin (H&E)‐stained tissue sections from four different levels of the lungs from all individuals (Figure 4B). The analysis revealed that the proportion of mice that had developed lung metastasis was significantly reduced in the group that received AAV‐mDNase I (6/10) vector compared to those that received the AAV‐null vector (8/10) (Figure 4C). These findings indicate that AAV‐mediated DNase I activity has the potential to counteract development of spontaneous breast cancer lung metastasis in mice.

### 3.7 Elevated levels of NETs in metastatic compared to local breast cancer in human cancer patients

To address the translational relevance of our findings and to confirm previous data using NE‐DNA complexes as a readout for increased levels of circulating NETs in metastatic breast cancer,^10^ we analyzed patient plasma using the H3R8cit ELISA, detecting citrullinated histone 3 bound to DNA, a biomarker for NETs.^20^ Plasma was sampled from patients with either local or metastatic breast cancer before initiation of treatment and from cured patients at 5‐year follow up. NET levels in plasma samples from cured patients were not different from those with local cancer (Figure 4D). In contrast, patients with metastatic cancer displayed significantly higher levels of NETs compared to those with local or cured cancer (Figure 4D), indicating a possible role for NETs as therapeutic targets and/or as biomarkers for metastatic disease.

## 4 DISCUSSION

In the current study we show that liver targeted AAV‐mediated systemic expression of murine DNase I can sustain high DNase activity in the circulation of mice over several months. Mice with breast cancer that received the AAV‐mDNase I vector had reduced urine levels of the biomarker NGAL compared to mice that received the AAV‐null control vector, confirming that the AAV‐mDNase I approach can suppress kidney dysfunction in a similar way as exogenously administered DNase I.^7^ Three mice in the AAV‐null control group had to be terminated 2 weeks before the experimental endpoint due to early development of large primary tumors. Despite this premature termination of three mice in the AAV‐null group, the proportion of PyMT mice that developed detectable metastases was significantly reduced in the AAV‐mDNase I group compared to the control group (AAV‐null). These findings demonstrate that AAV‐mediated expression of DNase I is a promising approach to suppress cancer‐associated systemic effects of breast cancer.

Five AAV‐based therapeutic strategies have been approved for clinical use, demonstrating the feasibility of this approach. The first therapeutic AAV was approved 2017 for the treatment of inherited retinal dystrophy,^23^ the second in 2019 for spinal muscular atrophy^24^ and the remaining three were all approved during 2023 for bleeding and neurological disorders.^25^, ^26^, ^27^ These AAV‐vectors are of serotypes AAV‐2, ‐5 or ‐9, displaying distinct organ tropism. The capsid used in the current study is KP1, an engineered chimeric capsid with robust tropism for murine liver as well as xenografted human hepatocytes.^18^ The liver‐specific promoter in the AAV‐mDNase I vector allows transgene expression selectively from the liver. As expected, analysis of DNase I expression in liver, kidney and heart from mice that received AAV‐mDNase I, compared to those injected with AAV‐null, showed increased expression only in liver and not in kidney, as expected owing to the liver‐specific promoter driving DNase I expression. DNase I was undetectable in heart (data not shown).

Importantly, we could not detect any liver toxicity due to the AAV‐administration or the AAV‐mediated expression of DNase I, judged by measurement of the liver enzymes ALT, AST and ALP in serum sampled 8 weeks after AAV administration. Toxicity could potentially arise from either the AAV itself or from the gene that is encoded by the AAV. When it comes to DNase I there is data available in support of that increased activity in the circulation is not harmful. DNase I is already in clinical use for cystic fibrosis patients in the form of an aerosol spray (Pulmozyme) since 1995.^28^ The inhaled DNase I helps dissolve mucus, shown to contain NETs^29^, ^30^ in the lungs of these patients. In patients with systemic lupus erythematosus (SLE), an autoimmune disease affecting multiple organs, an impaired ability to degrade NETs has been reported and associated with either anti‐DNase I antibodies,^31^ anti‐DNA antibodies^32^ or a mutation in the DNase I gene causing reduced expression levels.^33^ Therefore, a randomized trial in SLE patients was conducted with escalating doses of recombinant human DNase I, which was found to be well tolerated and without significant adverse events.^34^ In mice, genetic ablation of the two circulating DNases, DNase I and DNase I‐like 3, led to reduced degradation of NETs in sterile neutrophilia and septicemia, resulting in intravascular clots and organ damage.^35^ Altogether, these data indicate that too low levels of circulating DNase I might be more problematic than the opposite, and that DNases in the circulation are required for host protection against intravascular NETs and subsequent thrombosis.

Measuring liver enzymes in untreated PyMT mice and healthy littermates (FVB), we found indications of liver toxicity caused by the cancer itself. This will be important to take into account when analyzing AAV‐mediated liver toxicity in the PyMT mice and likely also in other preclinical models or patients. Moreover, this finding is in agreement with the ability of cancer to cause systemic effects on distant organs. Analysis of liver enzymes in serum from PyMT mice that received either AAV‐mDNase I or AAV‐null will reveal if enhanced serum DNase I activity could counteract also this type of cancer‐induced liver toxicity.

A role for NETs in promoting breast cancer invasiveness and metastasis via several different mechanisms have previously been reported.^9^, ^10^, ^11^, ^12^, ^13^, ^14^, ^15^ Published data include both in vitro and in vivo studies, using experimental metastasis models either injected directly in the circulation or in the mammary fat pad, resulting in spontaneous formation of lung metastases. The ability of DNase I administration to counteract metastasis has, however, not been addressed in a completely spontaneous model—from primary tumor development to established lung metastases—until now, which is a strength of the current study. A limitation of our study, as well as other preclinical studies addressing the role of NETs in breast cancer metastasis, is that lung is the only metastatic site of significance. In breast cancer patients, however, the most common site for development of metastases is the skeleton, then lung, lymph nodes, liver and brain. If NETs have a distinct role in metastasis to different sites is currently unknown. The plasma samples analyzed for NETs in the current study were derived from patients with metastatic disease in various organs (as described in the methods section), not only in lung.

The species‐specific approach for administration of DNase I is essential to avoid neutralization by the immune system. This is especially important when long‐term treatment and repeated injections are needed, for example to address effects on metastasis. We clearly show that this type of treatment is not feasible with bovine DNase I due to generation of anti‐bovine DNase I antibodies. We believe that the AAV‐mDNase I vector that we have now developed and characterized can become an important tool for preclinical studies that require long‐term DNase I treatment. Another aspect of the AAV‐based approach is that frequent injections will not be needed, which is an advantage both from a preclinical and clinical perspective. Likely it will also enable a more stable level of DNase I in the circulation without fluctuations. The duration of the murine DNase I activity in serum after a single injection was at least 5 months, most likely significantly longer. Exactly how long the AAV‐mediated DNase I expression lasts, particularly in humans, remains to be addressed. In the current study we could not analyze this in a conclusive manner since two mice did not survive until the experimental endpoint; one was sacrificed due to signs of decreased well‐being and one spontaneously died. If this spontaneous death had any connection to the AAV‐mDNase I administration is currently not known. However, spontaneous deaths in the FVB colony occur at a low frequency also in untreated individuals.

Another potential therapeutic strategy to reduce the level of intravascular NETs could be to use inhibitors of the enzyme peptidyl arginine deiminase 4 (PAD4). PAD4 is expressed by neutrophils and performs citrullination of arginine residues on histones, thereby reducing their positive charge and the strength of the interaction with DNA.^36^ Knockout of PAD4 in mice has been demonstrated to reduce formation of NETs.^37^, ^38^ A therapeutic advantage of PAD4 inhibition over DNase I administration could be that formation of NETs is prevented, in contrast to DNase I‐induced degradation of already formed NETs. A drawback could instead be that formation of NETs has also been reported to be independent of PAD4 activity. DNase I treatment on the other hand would target NETs regardless of the mechanism by which they were formed. Moreover, there is always a risk for unspecific effects that could not be predicted when using chemical drugs, while the specificity of DNase I is well described.

In conclusion, based on the data in the current study, we believe that this AAV‐based approach to enhance DNase I activity in the circulation constitutes a promising therapeutic option to counteract systemic effects of cancer.

## AUTHOR CONTRIBUTIONS

M.H., K.V. and A‐K.O. conceived and designed the research; M.H., K.V., J.C., S.N., F.S. and A‐K.O. performed the research and acquired the data; J.M., H.M., C.A.M., G.T. and V.T. provided essential samples or reagents; M.H., K.V., J.M., H.M., C.A.M., G.T., V.T. and A‐K.O. analyzed and interpreted the data. All authors were involved in drafting and revising the manuscript.

## FUNDING INFORMATION

This study was supported by The Swedish Cancer Society (201283 PjF) and The Swedish Research Council (2023‐02904) to AKO.

## DISCLOSURES

CAM has a financial interest in Sphere Gene Therapeutics, Inc., Chameleon Biosciences, Inc., and Skylark Bio, Inc., companies developing gene therapy platforms. CAM consults for and is a member of the scientific advisory board of CLS Therapeutics. CAM's interests were reviewed and are managed by MGH and Mass General Brigham in accordance with their conflict‐of‐interest policies.

## Supporting information


Data S1.

## Data Availability

The data that support the findings of this study are available in the materials and methods, results, and/or supplemental material of this article.
